# Early-Life Adversity and Epigenetic Aging: Findings from a 17-Year Longitudinal Study

**DOI:** 10.3390/biom15060887

**Published:** 2025-06-18

**Authors:** Emily Barr, Maude Comtois-Cabana, Andressa Coope, Sylvana M. Coté, Michael S. Kobor, Chaini Konwar, Sonia Lupien, Marie-Claude Geoffroy, Michel Boivin, Nadine Provençal, Nicole L. A. Catherine, Jessica K. Dennis, Isabelle Ouellet-Morin

**Affiliations:** 1 Faculty of Health Sciences, Simon Fraser University, Burnaby, BC V5A 1S6, Canada; ebarr@sfu.ca (E.B.); maude.comtois-cabana@umontreal.ca (M.C.-C.); andressa_coope@sfu.ca (A.C.); nadine_provencal@sfu.ca (N.P.); nicole_catherine@sfu.ca (N.L.A.C.); 2 BC Children’s Hospital Research Institute, Vancouver, BC V5Z 4H4, Canada; msk@cmmt.ubc.ca (M.S.K.); ckonwar@bcchr.ca (C.K.); 3 Centre for Molecular Medicine and Therapeutics, BC Children’s Hospital, Vancouver, BC V5Z 4H4, Canada; 4 Department of Psychology, University of Montreal, Montreal, QC H3C 3J7, Canada; sonia.lupien@umontreal.ca; 5 Department of Social and Preventive Medicine, University of Montreal, Montreal, QC H3N 1X9, Canada; sylvana.cote.1@umontreal.ca; 6 Department of Medical Genetics, University of British Columbia, Vancouver, BC V6T 1Z3, Canada; 7 Department of Educational & Counselling Psychology, McGill University, Montreal, QC H3A 1X1, Canada; marie-claude.geoffroy@mcgill.ca; 8 McGill Group for Suicide Studies, McGill University, Montreal, QC H3A 0G4, Canada; 9 School of Psychology, Laval University, Quebec City, QC G1V 0A6, Canada; michel.boivin@psy.ulaval.ca; 10 School of Criminology, University of Montreal, Montreal, QC H3T 1J4, Canada; 11 Research Centre of the Montreal Mental Health University Institute, Montreal, QC H1N 3M5, Canada

**Keywords:** aging, DNA methylation, epigenetic aging, early-life adversity, stress sensitization

## Abstract

Youth exposed to early-life adversity (ELA) are at greater risk for poorer physical and mental health outcomes in adolescence and adulthood. Although the biological mechanisms underlying these associations remain elusive, DNA methylation (DNAm) has emerged as a potential pathway. DNAm-based measures of epigenetic age have been associated with ELA, indicating accelerated aging. According to the stress sensitization hypothesis, prenatal adversity may further heighten sensitivity to subsequent stressors in childhood and adolescence. This study examined the associations between ELA and six epigenetic aging measures, considering both the timing of adversity and the participant’s sex. Data were drawn from the Quebec Longitudinal Study of Child Development, with two cumulative indices of ELA derived from prospectively collected data: the Perinatal Adversity and the Child and Adolescent Adversity indices. Higher Perinatal Adversity scores were associated with accelerated DunedinPACE scores. No significant associations were found between ELA and the other epigenetic clocks, nor did we find support for the stress sensitization hypothesis—though a sex-specific trend emerged among girls. The findings suggest that DunedinPACE may be more sensitive to variations in ELA than other clocks. Future research should systematically investigate sex-dimorphic associations between ELA and epigenetic aging, with particular attention to the impact of perinatal adversity.

## 1. Introduction

Early-life adversity (ELA) encompasses a range of potentially detrimental experiences, traumas, or stressors that occur during childhood and adolescence. Common examples of ELA include household dysfunction, family socioeconomic hardship, harsh parenting, and neighborhood violence [1,2]. Importantly, ELA is a cumulative concept at its core; it encompasses multiple forms of experiences, severity, and extent of exposure, occurring within and outside the family environment, and arising at any point from the prenatal period to the end of adolescence [3]. Longitudinal studies have shown that ELA robustly predicts physical, socioemotional, and behavioral problems later in life [4,5,6]. Despite ELA’s known risk for health and socioemotional functioning, the mechanisms underlying these associations remain poorly understood.

A common hypothesis suggests that ELA may induce persistent changes in physical and mental health by altering neurobiological stress systems designed to help the child cope with unpredictable, uncontrollable, and threatening environments [3,4,7]. For instance, studies have shown that children exposed to different levels of ELA have a disrupted pattern of hypothalamic–pituitary–adrenal (HPA) axis activity in both basal conditions (e.g., circadian rhythm [8,9]) and stressful conditions (e.g., magnitude and length of activation and recovery [10,11,12]). However, the strength and direction of these associations vary across studies. This inconsistency may stem from differences in the types of ELA considered, as well as a lack of attention to the co-occurring and cumulative effects of multiple adverse experiences [13]. Simultaneously accounting for multiple indicators has proven to be a promising approach. For example, Ouellet-Morin et al. [14] created a cumulative index of ELA that spanned several stressors within and beyond the family environment, measured from infancy to mid-adolescence. This index included eight indicators such as socioeconomic status, harsh parenting, and peer victimization, and was found to be non-linearly associated with hair cortisol concentration measured in late adolescence. Notably, only one indicator, neighborhood dangerousness, predicted hair cortisol concentration on its own, providing further support that cumulative indices of ELA better predict changes in stress systems than independent indicators. 

The mechanisms by which ELA affects later health are complex and involve several biological processes [15]. Epigenetic alterations, particularly DNA methylation (DNAm), have been proposed to take part in the associations between ELA, HPA axis activity, and subsequent health [16,17]. DNAm refers to the addition of a methyl group to specific sites in the genome where cytosine and guanine meet along the phosphate backbone, called CpG sites. However, these CpG sites can gain or lose a methyl group, potentially activating or inactivating genes and altering gene function across the life course. DNAm can both be affected by and induce changes in HPA axis activity, creating recursive loops that may perpetuate and exacerbate the impact of ELA on later functioning [18,19]. This emphasizes DNAm as a critical focus for understanding how adversity becomes embedded and influences vulnerability to later stress [4,16,20]. Clarifying whether DNAm serves as a mediating pathway between ELA and later health may inform targeted prevention strategies for individuals with a history of ELA [21,22,23].

Most studies to date have examined the association between ELA and DNAm using a candidate gene approach, focusing on DNAm near or within genes previously associated with ELA and/or relevant health outcomes [17]. Although this approach may be valuable, the greater part of the epigenome is overlooked, thus missing the opportunity to identify new loci of interest. Moreover, previous studies targeting the methylation of genes implicated in the regulation of stress, emotions, and behaviors, such as NR3C1, FKBP5, and SLC6A4, have yielded inconsistent findings with ELA experiences [17]. The potential partial overlap between candidate genes and other yet unidentified genes may impact the reproducibility of findings drawn from candidate genes vs. epigenome-wide approaches [24]. Conversely, epigenome-wide association studies comprise close to 1 million CpG sites depending on the array, exponentially increasing the number of tests and the possibility of false-positive and -negative findings (if the correction criteria are overly liberal or conservative, respectively). Adopting a more biologically informed approach, many studies elected to use polyepigenetic scores [17]. These scores encompass DNAm information across several CpG sites of interest collated into single scores, such as epigenetic clocks [25,26]. This approach enables the examination of multiple sites that may be more uniformly affected by ELA, extending beyond previously identified candidate genes, while reducing the number of tests by avoiding a full epigenome-wide analysis.

Epigenetic clocks estimate aging based on DNAm patterns, which can deviate from their chronological age. Epigenetic ages that exceed an individual’s chronological age have been linked with poorer health outcomes later in life, ranging from cognitive functioning [27] to high risk of cancer [28]. The first generation of epigenetic clocks, including the Horvath Pan-tissue Clock, focused on CpG sites that related solely to chronological age [25,29]. These clocks were then improved to encompass various tissues, such as the Skin and Blood Clock, and age ranges, such as with the Pediatric Buccal (PedBE) Clock [26,30]. The PedBE enabled researchers to shift the focus from aging-related decline to measuring developmental progress, offering a new perspective on what epigenetic clocks can capture. The focus then shifted from optimizing epigenetic clocks for chronological age to capturing the processes of biological aging and mortality more acutely, leveraging the information from biomarkers known to change with age and smoking, begetting a second generation of epigenetic clocks such as the PhenoAge and GrimAge Clocks [31,32]. To appropriately analyze first- and second-generation clocks, an epigenetic age acceleration variable ought to be derived to indicate the extent to which an individual’s epigenetic aging is accelerated or decelerated as compared to their chronological age [33]. Belsky et al. [34] adopted another approach by focusing on biomarkers that assess the integrity of physiological systems over time, both within and between individuals, resulting in DunedinPoAm, which was subsequently refined into DunedinPACE [35]. DunedinPACE reflects whether an individual is aging quickly or slowly, and requires no transformation into epigenetic age acceleration, thereby streamlining the analysis process and heralding a third generation of epigenetic aging measures. 

Epigenetic clocks have been, since the first generation, associated with numerous mental health outcomes linked to ELA, as well as experiences of ELA themselves. The original Horvath Pan-tissue Clock, for example, has been associated with psychiatric disorders [36,37,38,39], as well as experiences of childhood maltreatment [40,41] and socioeconomic disadvantage [42]. This trend has continued through the second generation, with GrimAge, for example, also being associated with psychiatric disorders [37,43], as well as experiences of ELA encompassing both violence [44] and socioeconomic disadvantage [45]. DunedinPACE has shown similar associations with these ELA indicators [43,46]. However, the inconsistent results are reported for all three generations of clocks [17]. We, and others [47], propose that the heterogeneity in these findings may partly stem from variations in the types of ELA examined—often in isolation—and differences in the timing of exposure (see Lupien et al. [48] for a similar assertion regarding HPA axis activity). Comparisons across studies focusing on different developmental periods, such as the prenatal period and adolescence, may yield misleading conclusions. Moreover, few studies have examined these associations using cumulative indices derived from prospectively and repeatedly collected indicators of adversity [37,49], which are expected to reveal more robust deviations in epigenetic clocks [17].

While ELA occurring during the pre- (before birth), peri- (around the time of birth), and postnatal (a few months after birth) periods—collectively referred to as the perinatal period—is often considered to exert the most enduring impact on neurobiological systems, increasing vulnerability for later problems [18,50,51], many studies overlooked their unique effects alongside later adversity [17]. Furthermore, emerging evidence suggests that ELA may increase sensitivity to future stressors through lasting neurobiological changes, as described by the stress sensitization hypothesis [52,53,54]. However, determining whether additive or synergic contributions better describe the associations between ELA and DNAm requires repeated measures across distinct periods of development (e.g., perinatal, childhood, and adolescence). To our knowledge, while this hypothesis has been explored in relation to DNAm and outcomes such as bipolar disorder and depression [52], it has not yet been tested with epigenetic clocks.

Finally, while sex differences are often reported for both ELA and epigenetic clocks, and the participant’s sex is typically included as a confounder in analyses, few studies have tested whether the direction or magnitude of these associations differed by sex [55]. This contrasts with recent evidence suggesting sex-specific differences between ELA and epigenetic aging, where adult males often show greater acceleration in epigenetic age compared to females who experienced similar adversity [56,57].

This study aimed to investigate the association between cumulative indices of ELA and six measures of epigenetic aging, each with its own strengths for use within our dataset. The specific objectives were threefold: (1) to test whether cumulative indices of ELA derived during the perinatal period and during childhood and adolescence (from 5 months to 15 years of age) were associated with the measures of epigenetic aging ascertained at 17 years of age; (2) to test the stress sensitization hypothesis, whereby the exposure to perinatal ELA exacerbated the strength of the associations between childhood and adolescent ELA and the measures of epigenetic aging; and (3) to examine whether these associations varied between males and females.

## 2. Materials and Methods

### 2.1. Cohort Description

The Quebec Longitudinal Study of Child Development (QLSCD) was initiated by the Institut de la Statistique du Quebec (ISQ) and included 2120 singleton children born between 1997 and 1998 in Quebec, Canada. The cohort has been described in detail elsewhere [58]. In brief, participants were recruited from all administrative regions of the province of Quebec, excluding First Nations’ Territories and Northern Quebec due to differences in institutional services and socio-cultural context. Families were invited to participate if the pregnancy lasted between 24 and 42 weeks, and if parents could speak French or English. The resulting participants were representative of the general population. Participants were followed annually or biennially from 5 months to the present. At age 17, 1150 youths still in contact with the survey and who lived in the province of Quebec were invited to provide a saliva sample for the measurement of DNA methylation. Ultimately, 773 (67.2%) of the invited youths provided a saliva sample. The QLSCD protocol was approved by the ISQ and the University of Montreal ethics committees, and informed consent forms (and the participating child’s assent when over 10 years of age) were obtained at each data collection.

### 2.2. Measures

#### 2.2.1. Perinatal Adversity Index

Information about adverse experiences during perinatal development was provided by parents via questionnaires when the participating children were 5 months old or retrieved from hospital records (birth weight and gestational time). Seven indicators of adversity were included, based on the cumulative index for adversity previously designed and implemented in the Maternal Adversity, Vulnerability, and Neurodevelopment (MAVAN) cohort [59]. These indicators were (i) low birth weight, (ii) pre-term birth, (iii) low socioeconomic status (SES), (iv) maternal smoking during pregnancy, (v) hospitalization in the first 5 months of life, (vi) maternal depression at 5 months postpartum, and (vii) poor family function. Due to the general low-risk nature of this population-based cohort, all items were dichotomized to reflect the presence (1) or absence of (0) a given adverse experience. (i) Low birth weight was recorded as <2500 g [60]. (ii) Pre-term birth was recorded as <37 weeks. (iii) Low SES was recorded as a <CAD 30,000 gross household annual income. (iv) Maternal smoking during pregnancy (yes/no) was derived from the maternal questionnaire item “Did you smoke during pregnancy?”. (v) Hospitalization in the first five months of life (yes/no) was derived from a parental report. (vi) Postnatal maternal depression (yes/no) was assessed with a shortened (13 items) version of the Center for Epidemiologic Studies Depression Scale (CES-D), which focused on depressive symptoms in the previous week. Mothers were scored on 16 items indicating clinically relevant depressive symptomatology (e.g., “I was bothered by things that usually don’t bother me”) on a Likert scale varying from 0 (rarely or none of the time (less than one day)) to 3 (most or all of the time (5–7 days)) [61]. (vii) Poor family functioning (yes/no) was assessed using the Family Functioning FNC Survey (Q1A-Q1M) [58]. Both parents independently completed the survey, which measured marital strain, as well as a low sense of cohesiveness and closeness in the first 5 months postpartum. The survey included 13 items measured on a 4-point Likert scale (e.g., “We express feelings to each other”; 1 (strongly agree) to 4 (strongly disagree)). If either parent had a score above the 90th percentile, the family was considered to have poor family functioning. The seven binary indicators were summed to derive the cumulative Perinatal Adversity index. Only three indicators had missing data (maternal smoking during pregnancy: n = 2; low SES: n = 1; and family function: n = 2). The missing data was replaced with the indicator’s most frequent response (0). Although the cumulative index theoretically ranged from 0 to 7, very few participants had scores above 3. We therefore collapsed our scores above 2 into a single group of 2+ to ensure the distribution was not too positively skewed for the analyses (0: 44.4%; 1: 32.0%; 2: 23.6%).

#### 2.2.2. Child and Adolescent Adversity Index

We derived the Child and Adolescent Adversity index as previously reported [14]. Briefly, the cumulative index included information from eight distinct indicators of socioeconomic and psychosocial adversity, all previously associated with poor health [22]. Indicators were collected prospectively and repeatedly from 5 months to 15 years of age. The cumulative index included the following indicators: (i) young motherhood, (ii) single-parent household, (iii) low SES, (iv) harsh and coercive parenting, (v) maternal depression, (vi) maternal alcohol consumption, (vii) peer victimization, and (viii) dangerous neighborhoods. (i) Young motherhood was collected at 5 months old and recorded as present if the mother was younger than 21 years of age when she had her first child, regardless of whether the first child was the child included in the QLSCD. (ii) Single-parent household was determined from maternal reports at any point during the child’s preschool years, which were collected up to six times between 5 months and 5 years. (iii) Low SES was reported by the parents, and this data was collected up to 11 times between 5 months and 15 years, which incorporated information about low family income (<CAD 30,000 per year), mothers’ and fathers’ education, and occupational prestige. (iv) Harsh and coercive parenting practices were assessed by maternal reports at up to 10 occasions between 2.5 and 15 years. These reports included 4–8 items, dependent on the child’s age, and referred to power-assertive behaviors manifested toward the child (i.e., “I have been angry with my child when he was particularly fussy”, from 0 (“Not at all what I did”) to 10 “(Exactly what I did”)). (v) Maternal Depression was reported by mothers 7 times, between 5 months and 13 years, according to the same questionnaire described for the Perinatal Adversity index. (vi) Maternal alcohol consumption was reported by mothers through questionnaires completed at up to 6 time points between 5 months and 15 years on an 8-point Likert scale ranging from “never” to “every day”. (vii) Peer victimization was reported by the participating youth at seven time points from age 6 to 15, utilizing the Self-Report Victimization Scale [62]. This scale includes seven items, encompassing the perceived occurrence of physical, verbal, relational, and cybervictimization, and was measured on a 3-point Likert scale from “never” to “often or very often”. (viii) Neighborhood dangerousness was reported by mothers at up to eight time points between 5 months and 15 years to measure a lack of security and cohesion, using five items from the Simcha-Fagan Neighborhood Questionnaire (i.e., “Around here, when there is a problem, neighbors get together to find a solution.”).

As previously reported [14], group-based growth mixture models used all available data from each indicator collected over time (excluding young motherhood) to estimate stable patterns of adversity exposure. The best fitting model for each indicator was selected based on fit and parsimony indices (e.g., Bayesian Information Criterion (BIC), Lo–Mendell–Rubin likelihood ratio test (LMR-LRT), and entropy estimates). Class membership then designated groups exposed to low (0), moderate (1), or high (2) levels of adversity according to each indicator. These scores were then summed across all eight indicators to create the Child and Adolescent Adversity index, varying between 0 and 13. As very few individuals scored 12 or higher, we collapsed our distribution between 0 and 11 to avoid the disproportionate weight of the higher scores in the regression analyses using all available data. Only individuals with complete data for the Child and Adolescent Adversity index (n = 696; 99.6%) were included for subsequent analyses.

### 2.3. DNA Methylation Quantification

Participants provided saliva samples using the Oragene Self-Collection Kit (DNA Genotek, Ottawa, ON, Canada). DNA purification was performed following the Beckman Coulter Genomics Laboratory protocol for the manual purification of DNA from 500 μL of Oragene/saliva samples using the Agencourt DNAdvance Kit (Beckman Coulter, Brea, CA, USA). DNA was quantified primarily using absorbance measured on the NanoDrop 8000 Spectrophotometer (Thermo Fisher Scientific, Waltham, MA, USA), and a subset of samples was tested with fluorescence (pico green) on the Qubit dsDNA BR Assay kit (Thermo Fisher Scientific, Waltham, MA, USA) to ensure the absorbance was in an acceptable range. Finally, bisulfite conversion was performed with the EZ-96 DNA Methylation Kit (Zymo Research, cat. No. D5004, Irvine, CA, USA), following the manufacturer’s protocol. Bisulfite DNA was quantified again using the absorbance method (for RNA), and 250 ng of bisulfite DNA from each sample was extracted with the EZ-96 DNA Methylation Kit (Zymo Research, Irvine, CA, USA) and then run on the Infinium MethylationEPIC v1.0 BeadChip (Illumina, San Diego CA, USA).

DNA methylation beta values were extracted and processed using R software (version 3.6.2), as well as with the minfi R package, version 1.54.1 [63]. Poor quality samples were initially flagged based on the ratio of the log2 unmethylated to methylated intensity of the control probes at a default cutoff of 10.5 and further confirmed by inspecting control probe intensities in the quality control report from minfi. To adjust for the different intensities of the two different probe types on the array, we used Noob normalization for dye-bias and background correction (the preprocessNoob command from the minfi package, version 1.54.1). We performed a sex check to confirm concordance between reported and inferred sex, based on XX/XY intensities, and removed mismatches. Further, probe filtering was performed based on published annotations [64,65] to remove probes that cross-react or cross-hybridize to multiple autosomal and sex probes, as well as probes that had a detection *p*-value greater than 0.01 in 25% of samples, indicating that probes were either too sensitive or not sensitive enough. Batch correction was not performed, as our normalization method effectively removed the most prominent batch effects. Overall, after probe and sample quality checks, 817,658 probes and 696 samples were retained for subsequent analyses.

#### Cell Type Estimation

As DNA methylation is cell type-specific, and saliva comprises multiple cell types including neutrophils, monocytes, and epithelial cells, we used DNA methylation-based cell type deconvolution in the R package EpiDISH version 2.24.0 [66] to infer the saliva cell types (Supplemental Figure S1).

### 2.4. Epigenetic Age Acceleration

To account for the different strengths and limitations of different epigenetic clocks, including their target age ranges, cell types, and generations, we measured epigenetic aging in the QLSCD with six different clocks. We used R (version 4.3.1) and adjusted the publicly available code to calculate all epigenetic age measures but one, the Horvath Pan-tissue Clock, which we calculated by submitting our data to an online portal. We included three measures of first-generation clocks, which were designed to measure chronological age: (1) Horvath Pan-tissue Clock [25]; (2) the Skin and Blood Clock [26]; and (3) the PedBE Clock [30]. We also included two measures of second-generation clocks, which improved epigenetic age derivation by incorporating phenotypic measures of aging: (4) PhenoAge [31] and (5) GrimAge [32]. Finally, we investigated a third-generation measure of epigenetic aging, (6) DunedinPACE [35], which expands on both prior generations by incorporating comprehensive measures of changes in physiological biomarkers for organ-system integrity over time. The first five clocks were regressed against chronological age to derive a measure of epigenetic age acceleration; this step was unnecessary for DunedinPACE, as it directly provides a measure of the “pace of aging” instead of an epigenetic age. For each clock and DunedinPACE, we also removed the confounding effect of cell type composition by deriving the residuals from regressions; therefore, epigenetic age acceleration was calculated with the regression against both chronological age and cell type. We used this approach rather than including cell type proportions as covariates due to high correlation with cell type across the epigenetic clocks, and a low covariation between cell type and ELA. In addition, the GrimAge Clock includes sex differences in its derivation, resulting in epigenetic ages heavily skewed by sex. As such, sex was also regressed from the GrimAge variable along with both chronological age and cell type, and this clock was excluded from testing the moderating role of sex (Supplemental Figure S2).

#### Assessment of Epigenetic Age Measure Performance

Pearson’s correlation coefficients (r) between our measures of epigenetic age and participant chronological age were measured for all five clocks, excluding DunedinPACE, as it does not measure age in years. Correlations were also measured between all predicted measures of epigenetic age. Given the homogeneity of chronological age in our cohort (mean = 17.22; SD = 0.27), we expected weaker correlations between each epigenetic age and chronological age compared to the correlations between the epigenetic aging measures. We calculated the mean absolute error and maximum absolute error (MAE and MaxAE) for each measure of epigenetic age and chronological age using the mae and maxae commands from the mlr3measures package [67].

### 2.5. Covariates

Race/ethnicity was self-reported by the parents. Individuals were considered non-White if at least one parent selected any option for race/ethnicity other than “White”. As our cohort was overwhelmingly White (93%), we dichotomized race/ethnicity as “White” vs. “non-White”. Although this dichotomization masks potentially important differences across non-White race/ethnicity people, it ensured that we maintained sufficient participants in each group. Information about tobacco, cannabis, and alcohol consumption within the year prior to saliva sample collection was provided by participants at age 17 on a 4-point Likert scale (“never” to “daily”). BMI at age 17 was also self-reported. Age at the time of sample collection was also included as a covariate in our final models of epigenetic age acceleration because even slight differences in age may confound the adversity–epigenetic age associations [33] (Supplemental Table S1).

### 2.6. Statistical Analyses

All statistical analyses were performed in R 4.3.1, and linear regressions used the base linear model (lm) function. Statistical significance was initially defined as a *p*-value < 0.05, and we subsequently applied the false discovery rate (FDR) < 0.10 for multiple testing correction. First, we examined the associations between our six measures of epigenetic age with Perinatal Adversity as well as with Child and Adolescent Adversity in unadjusted and adjusted models, with the latter including all covariates. Second, we tested the unique contribution of each index of ELA, and the hypothesized sensitization influence of Perinatal Adversity on the association between Child and Adolescent Adversity and the measures of epigenetic clocks. Finally, we examined the moderating influence of sex in these associations by including an interaction term between sex and each adversity index separately and together (i.e., triple interaction). Significant interactions were decomposed by the moderator (sex and/or Perinatal Adversity) to depict their simple slopes. Effect sizes were measured using standardized betas and Cohen’s f^2^ for full models (where f > 0.02 indicates a small effect, f > 0.15 is a medium effect, and f > 0.35 is a large effect) (Supplemental Table S2).

## 3. Results

### 3.1. Exposure to Adversity Was Low Overall and Differed by Sex

Of the 773 participants who provided a saliva sample for DNAm quantification, 39 did not have enough DNA for bisulfite conversion, 35 were dropped due to poor probe intensity in the array data, and 3 were dropped due to incomplete adversity data for both Perinatal and Child and Adolescent Adversity. The analyses were carried out on 696 participants (385 females, 55%). The prevalence of Perinatal Adversity in the cohort was low and similar across sexes (Table 1).

In contrast, a greater proportion of the cohort experienced some types of Child and Adolescent Adversity, although, on average, the overall score was low to moderate (Table 2). Compared to females, males experienced higher levels of peer victimization, lower family SES, and harsher and more coercive parenting. This difference translated into a statistically significantly higher cumulative score in males compared to females.

The two indicators of adversity are moderately and significantly correlated, according to Kendall’s correlation (τ = 0.28).

### 3.2. Measures of Epigenetic Age Among Adolescents

As most measures of epigenetic aging are trained on individuals in adulthood, and more specifically, middle-aged individuals, we examined their association with chronological age in our adolescent cohort. Although we report the correlation estimates between each clock and chronological age, the limited age range (16.8–18.1 years) of the present sample greatly limited this otherwise widely used validation technique. Unsurprisingly, all measures showed low correlation with chronological age, with only the Skin and Blood Clock (r = 0.09) and GrimAge (r = 0.08) reaching significance. Comparing correlations between clocks, DunedinPACE and PhenoAge had the highest correlation (r = 0.67), followed by DunedinPACE and GrimAge (r = 0.27). The rest of the clocks were weakly correlated (r > 0.15), except for the Pediatric Clock, which did not significantly correlate with any of our measures or chronological age. We also included mean and maximum errors, which were low for all clocks (MAE < 5.5 years of error) except for GrimAge, which had an MAE > 10 years (Supplemental Table S3). This indicated that, while there was limited overlap between the clocks, the error in clock calculations was small across all but the GrimAge Clock.

### 3.3. Only Perinatal Adversity Is Associated with Epigenetic Age Acceleration

Regression analyses between measures of epigenetic age and adversity indices revealed that DunedinPACE was the only measure of epigenetic age that was significantly associated with the Perinatal Adversity index, even after adjusting for confounders (β = 0.079, *p*-value = 0.045, Cohen’s f^2^ = 0.098) (Table 3). This finding did not survive multiple testing correction, though.

For the Child and Adolescent Adversity index, the unadjusted models indicated associations with two measures of epigenetic aging at a trend level: PedBE (β = 0.065, *p*-value = 0.085, Cohen’s f^2^ = 0.0043) and DunedinPACE (β = 0.066, *p*-value = 0.083, Cohen’s f^2^ = 0.0043). However, none of these measures of epigenetic age were significantly associated with the Child and Adolescent Adversity index after controlling for the covariates (Table 3).

**Figure 1:**
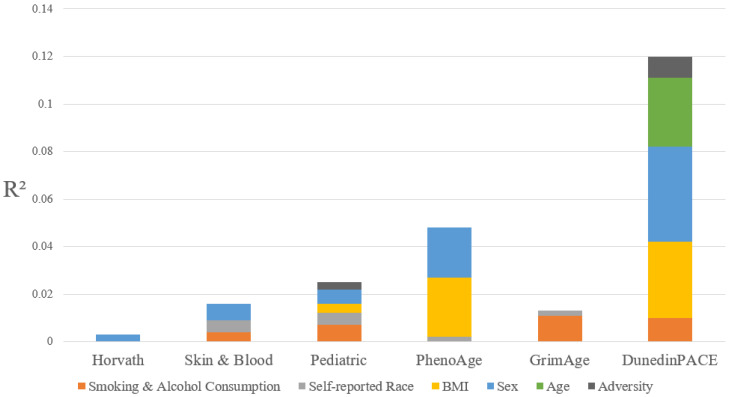
Portion of variance in epigenetic age measures explained by confounders and adversity indices in the QLSCD ^a^. Notes: Smoking & Alcohol Consumption includes tobacco, cannabis, and alcohol consumption. Adversity includes both Perinatal Adversity and Child and Adolescent Adversity indexes. All clocks, excluding DunedinPACE, were adjusted to give epigenetic age acceleration by taking the residuals of the calculated age regressed on chronological age. All age measures were additionally regressed against cell type proportions before multivariable modeling. The GrimAge Clock was also adjusted for sex before multivariable modeling. ^a^ Data were compiled from the final master file of the Québec Longitudinal Study of Child Development (1998–2015), ©Gouvernement du Québec, Institut de la statistique du Québec.

We performed forward regression to more precisely describe the relative proportion of variance in each measure of epigenetic age explained by the adversity indices and each confounder (i.e., race/ethnicity, tobacco, cannabis, and alcohol consumption, BMI, age, and sex) (see Figure 1). DunedinPACE had the highest explained variance (12.0%), as well as the most variance explained by adversity indices (1%). In addition, DunedinPACE was the only measure of epigenetic age with variance explained by chronological age (3%) in this otherwise age-homogeneous sample. As expected, tobacco, cannabis, and alcohol consumption, BMI, and sex each contributed to the explained variance across the clocks, even though the magnitude of these effects varied.

### 3.4. Limited Evidence for the Stress Sensitization Hypothesis

Before formally testing the stress sensitization hypothesis, we first included both Perinatal (β = 0.065, *p*-value = 0.12) and Child and Adolescent Adversity (β = 0.045, *p*-value = 0.29) indices (additive model, Cohen’s f^2^ = 0.10) to evaluate their unique contributions. We found no main significant associations, including for DunedinPACE, suggesting that both adversity indices were weakly associated with a common portion of DunedinPACE variance (Table 4). We then tested the stress sensitization hypothesis by adding an interaction term to the two adversity indices. A significant association between the interaction term and epigenetic age acceleration would lend support to this hypothesis. However, none of the interactions were significant.

### 3.5. Epigenetic Age Differs by Sex, but Evidence of Sex Moderation Is Limited

Significant sex differences emerged in Welch’s two-sample *t*-tests for two clocks, PhenoAge (*t* = −4.56, *p* = < 0.001) and DunedinPACE (*t* = −5.79, *p* = < 0.001), whereby males had a lower PhenoAge and DunedinPACE than females. Regression analyses including interaction terms between sex and each measure of ELA did not detect sex-dimorphic associations for the Perinatal or Child and Adolescent Adversity indices alone. However, a trend for a triple interaction emerged for DunedinPACE (β = 0.073, *p*-value = 0.072, Cohen’s f^2^ = 0.10), suggesting that a distinct pattern of stress sensitization may be present between males and females (Table 5).

**Figure 2:**
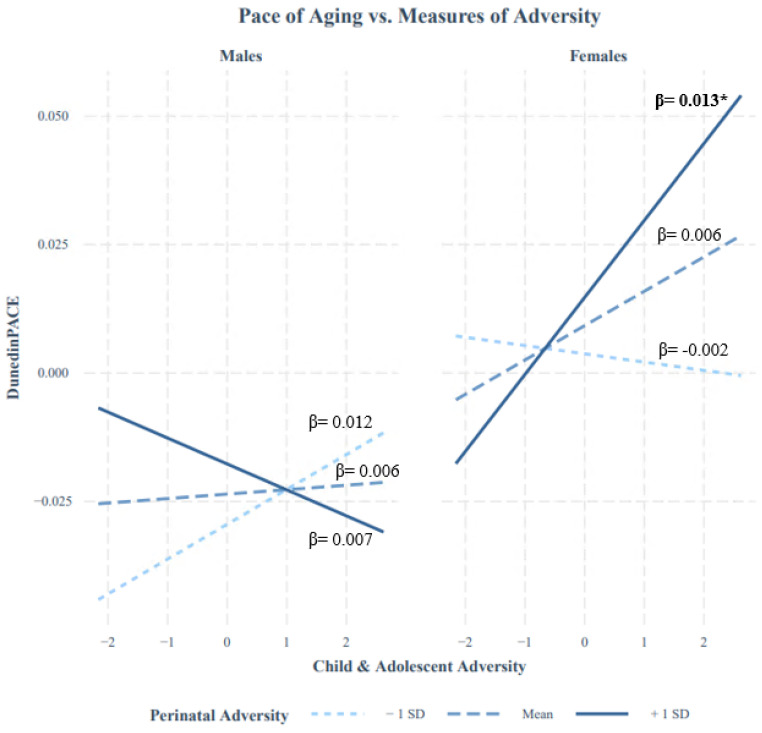
Model-predicted associations between Child and Adolescent Adversity and DunedinPACE for male (**left**) and female (**right**) participants with Perinatal Adversity scores at ±1 SD above the mean in the QLSCD ^a^. Bold values with * are significant at *p* < 0.05. Perinatal Adversity and Child and Adolescent Adversity indices were mean-centered to investigate simple slopes. ^a^ Data were compiled from the final master file of the Québec Longitudinal Study of Child Development (1998–2015), ©Gouvernement du Québec, Institut de la statistique du Québec.

To explore this tentative signal, we tested whether distinct patterns of stress sensitization could have been hidden (i.e., canceled out) for males and females. No robust indication of significant interactions between Perinatal Adversity and Child and Adolescent Adversity emerged for males (β = −0.068, *p*-value = 0.28) or females (β = 0.081, *p*-value = 0.13). Figure 2 illustrates the simple slopes between Child and Adolescent Adversity and the DunedinPACE scores according to ±1 standard deviation of the mean of the Perinatal Adversity, for males and females separately. Notably, however, opposite patterns of moderation were noted: females exposed to higher levels of Perinatal Adversity tended to have higher DunedinPACE scores in association with higher levels of Child and Adolescent Adversity (β = 0.15, *p* = 0.042), whereas no associations were detected at or below the sample’s mean of Perinatal Adversity (mean: β = 0.06, *p* = 0.81; −1SD: β = −0.02, *p* = 0.25). None of the simple slopes were significant in males. As post hoc analyses were conducted based on a trend for significance, interpretations of the sex-moderated stress sensitization effect should be approached with caution.

## 4. Discussion

This study examined the association between two cumulative indices of ELA and six measures of epigenetic aging in a cohort of 17-year-old youth. This allowed us to investigate whether Prenatal vs. Child and Adolescent adversity is differently related to epigenetic aging. We also formally tested the stress sensitization hypothesis and whether these associations differed by sex. Our findings provided limited evidence for these hypotheses. However, a few isolated findings suggested that the DunedinPACE measure may be more sensitive than the other epigenetic clocks at detecting such effects.

DunedinPACE was the only measure of epigenetic aging that captured, albeit inconsistently, variations in ELA in our cohort of adolescents. Furthermore, it was the only measure that captured chronological age as a measure that influenced variance in our data. This distinct pattern of findings may echo the differences in how each measure of epigenetic aging was derived. Unlike other epigenetic clocks, DunedinPACE was built from data collected repeatedly in the same individuals to capture physiological changes both within and between individuals. In contrast, other epigenetic clocks were derived from inter-individual differences in chronological age or changes in biomarkers at a single time point [35]. DunedinPACE may thus be better suited to capture deviations induced by ELA over time, especially earlier in development, given its focus on physiological systems related to accelerated aging. Also, both our sample and the cohort from which the DunedinPACE measure was derived are population-based, with relatively few individuals exposed to very high levels of adversity. In community-based samples like ours, variation in socioeconomic deprivation has been associated with DunedinPACE [34,35,68,69]. Raffington et al. [46], for instance, have reported an association between a composite measure of socioeconomic disadvantage—including a combination of household income, parental education, occupational prestige, and measures of neighborhood disadvantage—and DunedinPoAm, the precursor to DunedinPACE. Consistent with our findings, they found no associations between their measures of deprivation and the Horvath Pan-tissue Clock, the Hannum Clock, PedBE, PhenoAge, and GrimAge. Our null findings for Child and Adolescent Adversity and these other measures of epigenetic age are thus in line with much of the literature focused on community-based cohorts [41,42,49,70]. In contrast, associations with ELA in other epigenetic clocks appear to be more robustly detected in at-risk samples, comprising those exposed to greater levels of adversity [47,71] or those specifically associated with changes in epigenetic age over time [72].

Contrary to our expectations, our findings did not offer robust support for the stress sensitization hypothesis. We did, however, find that female participants who experienced higher levels of Perinatal Adversity had higher DunedinPACE scores when they had also experienced higher levels of Child and Adolescent Adversity than their peers with lower or no Perinatal Adversity. This could suggest that females may be prone to be more negatively affected by re-exposure to adversity. Although the stress sensitization hypothesis has been scarcely examined in relation to measures of DNAm, there is support for this sex-specific finding in both stress sensitization research and studies investigating overall stress [73,74]. A 2017 meta-analysis by Bunea et al. [53] found that women with a history of ELA required less exposure to recent stress, as measured by cortisol levels, to trigger post-traumatic stress disorder compared to their male counterparts. Stroud (2020) [75] provided an in-depth review of Post’s model of stress sensitization, summarizing findings across the literature and discussing the model’s strengths and limitations. Notably, sex emerged as a key factor influencing stress sensitization outcomes, with sex-specific effects observed depending on pubertal stage.

Sex differences in methylation are also well documented, pointing to higher epigenetic age in males in comparison to females [55,76,77]. However, we found higher measures of epigenetic aging in females than in males using both PhenoAGE and DunedinPACE, both of which derive epigenetic age from physiological biomarkers of aging. A possible explanation for this difference in our study compared to the existing literature is the age of our participants (17 years) compared to previous studies conducted with adult samples [31]. Both genetic and epigenetic mechanisms interact with hormone activity, changing during puberty. At 17 years of age, neither males nor females have typically reached full pubertal maturation; however, females generally tend to be more advanced in the pubertal process than males [78]. The PedBE Clock included few individuals over age 15 and shows increasing prediction error with age, limiting its accuracy in adolescence, as acknowledged by its developers [30]. Its good performance at age 10 in a subsample of our cohort [70] further supports these age-related limitations in its design. It remains uncertain whether measuring epigenetic aging a decade later (e.g., in one’s twenties) may provide additional insights, but it would enable the analysis of intra-individual changes in epigenetic aging over time and document, if present, any shift in mean levels between males and females [71,76,79]. Nonetheless, these preliminary findings underscore the importance of systematically investigating and reporting sex differences in epigenetic aging in future studies.

Our study had several limitations. First, we measured DNAm in saliva, and while each measure of epigenetic aging has been adjusted for use in saliva by controlling for cell type proportions, three measures were originally derived for use in blood and may not be optimized for use in other tissues [31,32,35]. Second, our study sample was 17 years old at the time of DNAm collection, and each measure of epigenetic aging used in this study was derived and optimized for use in age groups younger than (PedBE, optimized for children prior to adolescence) or older than (remaining epigenetic aging measures) our sample. Third, our cohort included youth primarily of self-reported White race/ethnicity, limiting the generalizability of our results. Fourth, levels of exposure to ELA were relatively low in our cohort; most parents had completed a high school diploma or higher, and the average household income exceeded the national poverty cutoff. Given that exposure to threat and abuse has more frequently been associated with epigenetic age acceleration than experiences of neglect and deprivation, it is possible that distinct findings could be uncovered in higher-risk cohorts [40,46,47].

## 5. Conclusions

This study sought to expand upon the existing literature investigating the relationship between ELA and epigenetic aging by incorporating numerous clocks and two cumulative measures of ELA and by accounting for the timing of ELA and the potential impact of sex differences in adolescence. Strengths of our study include our use of comprehensive, prospectively, and repeatedly collected indicators of adversity. Future studies would benefit from examining these associations in higher-risk samples and according to a dimensional approach of adversity, such as exposure to socioeconomic vs. relational adversity or threat vs. abuse experiences of maltreatment (1). Overall, exposure to ELA was low in our cohort, and we found minimal evidence of associations between ELA and epigenetic aging. Nonetheless, of all the measures of epigenetic aging tested, DunedinPACE captured the most variation in ELA, and we found some evidence that female children exposed to more adversity from before birth to adolescence experienced greater epigenetic aging measured by DunedinPACE. Future studies should systematically examine sex differences in DNAm in response to ELA.

## Figures and Tables

**Table 1:** Summary of each indicator included in the Perinatal Adversity index according to the total sample and by sex in the QLSCD ^a^.

	Total (n = 696)	Female (n = 385)	Male (n = 311)	Mean Differences
	n (%) or Mean (SD)	n (%) or Mean (SD)	n (%) or Mean (SD)	Chi-Square Test (*p*-Value) or *t*-test (*p*-Value)
Specific Indicators				
Low Birth Weight (<2500 g)	26 (3.7%)	14 (3.6%)	12 (3.9%)	6.8 × 10^−31^ (1.00)
Pre-Term Birth (<37 weeks)	92 (13.2%)	44 (11.4%)	48 (15.4%)	2.07 (0.15)
Low SES (<CAD 30,000)	156 (22.4%)	89 (23.1%)	67 (21.5%)	0.16 (0.69)
Maternal Smoking (Yes)	147 (21.1%)	82 (21.3%)	65 (20.9%)	0.001 (0.97)
Hospital Admissions (First 5 Months)	80 (11.5%)	39 (10.1%)	41 (13.2%)	1.29 (0.26)
Maternal Depression (CESD > 16)	31 (4.5%)	20 (5.2%)	11 (3.5%)	0.76 (0.39)
Poor Family Functioning	61 (8.8%)	35 (9.1%)	32 (8.4%)	0.04 (0.84)
Total Score				
Perinatal Adversity (Range 0–2)	0.74 (0.79)	0.73 (0.79)	0.77 (0.78)	0.73 (0.46)

Notes: There were no mean differences by sex for any indicator included in Perinatal Adversity. ^a^ Data were compiled from the final master file of the Québec Longitudinal Study of Child Development (1998–2015), ©Gouvernement du Québec, Institut de la statistique du Québec.

**Table 2:** Summary of each indicator included in the Child and Adolescent Adversity variable for the total sample and by sex in the QLSCD ^a^.

	Total (n = 696)	Female (n = 385)	Male (n = 311)	Mean Differences
	n (%) or Mean (SD)	n (%) or Mean (SD)	n (%) or Mean (SD)	Chi-Square Test (*p*-Value) or *t*-test (*p*-Value)
Specific Indicators				
Young Motherhood Yes	133 (19.1%)	69 (17.9%)	64 (20.6%)	0.62 (0.43)
Single-Parent Household Yes	90 (12.9%)	46 (11.9%)	44 (14.1%)	0.56 (0.46)
Socioeconomic Status (Low vs.) Medium High	283 (40.7%) 174 (25.0%)	141 (36.6%) 106 (27.5%)	142 (45.7%) 68 (21.9%)	6.23 (**0.044**)
Coercive Parenting (Low vs.) Medium High	327 (47.0%) 84 (12.1%)	173 (44.9%) 36 (9.35%)	154 (49.5%) 48 (15.4%)	10.82 (**0.004**)
Maternal Depression (Low vs.) Medium High	285 (40.9%) 76 (10.9%)	152 (39.5%) 42 (10.9%)	133 (42.7%) 34 (10.9%)	0.85 (0.66)
Maternal Alcohol Consumption (Low vs.) Yes	143 (20.5%)	76 (19.7%)	67 (21.5%)	0.24 (0.62)
Peer Victimization (Low vs.) Medium High	390 (56.0%) 84 (12.1%)	199 (51.7%) 36 (9.35%)	191 (61.4%) 48 (15.4%)	21.66 (**<0.001**)
Dangerous Neighborhood (Low vs.) Medium High	561 (80.6%) 42 (6.03%)	306 (79.5%) 27 (7.01%)	255 (82.0%) 15 (4.82%)	1.52 (0.47)
Total Score				
Child and Adolescent Adversity (Range 0–11)	4.97 (2.30)	4.76 (2.31)	5.23 (2.26)	2.72 (**0.007**)

Notes: Mean differences by sex with a *p* < 0.05 are marked in bold. ^a^ Data were compiled from the final master file of the Québec Longitudinal Study of Child Development (1998–2015), ©Gouvernement du Québec, Institut de la statistique du Québec.

**Table 3:** Adjusted associations between both adversity indices and measures of epigenetic age acceleration in the QLSCD ^a^.

Model	Horvath	Pediatric Clock	Skin and Blood Clock	PhenoAge	GrimAge	DunedinPACE
	Estimate (*p*val)	Estimate (*p*val)	Estimate (*p*val)	Estimate (*p*val)	Estimate (*p*val)	Estimate (*p*val)
Perinatal Adversity	−0.00025 (0.99)	0.024 (0.55)	0.020 (0.62)	0.023 (0.57)	0.0033 (0.94)	**0.079 (0.045)**
Child and Adolescent Adversity	−0.035 (0.41)	0.056 (0.17)	−0.0016 (0.97)	0.0037 (0.93)	0.029 (0.50)	0.064 (0.12)

Notes: Bold values are significant at *p* < 0.05. All clocks, excluding DunedinPACE, were adjusted to give epigenetic age acceleration by taking the residuals of the calculated age regressed on chronological age. All age measures were additionally regressed against cell type proportions before multivariable modeling. The GrimAge Clock was also adjusted for sex before multivariable modeling. All models were controlled for self-reported race/ethnicity, tobacco smoking, cannabis smoking, alcohol consumption, BMI, age, and sex. Estimate refers to standardized regression coefficients. ^a^ Data were compiled from the final master file of the Québec Longitudinal Study of Child Development (1998–2015), ©Gouvernement du Québec, Institut de la statistique du Québec.

**Table 4:** Associations between adversity variables and epigenetic age acceleration, testing adversity timing in the QLSCD ^a^.

Model		Horvath	Pediatric Clock	Skin and Blood Clock	PhenoAge	GrimAge	DunedinPACE
		Estimate (*p*val)	Estimate (*p*val)	Estimate (*p*val)	Estimate (*p*val)	Estimate (*p*val)	Estimate (*p*val)
Perinatal Adversity + Child and Adolescent Adversity							
	Perinatal Adversity	0.011 (0.79)	0.0070 (0.87)	0.022 (0.88)	0.024 (0.58)	−0.0050 (0.91)	0.065 (0.12)
	Child and Adolescent Adversity	−0.039 (0.38)	0.055 (0.21)	−0.0067 (0.61)	−0.0022 (0.96)	0.027 (0.55)	0.045 (0.29)
Perinatal Adversity*Child and Adolescent Adversity							
	Perinatal Adversity*Child and Adolescent Adversity	−0.029 (0.48)	0.0077 (0.85)	−0.020 (0.62)	−0.054 (0.18)	0.010 (0.80)	0.027 (0.49)

Notes: All clocks, excluding DunedinPACE, were adjusted to give epigenetic age acceleration by taking the residuals of the calculated age regressed on chronological age. All age measures were additionally regressed against cell type proportions before multivariable modeling. The GrimAge Clock was also adjusted for sex before multivariable modeling. All models were controlled for self-reported race/ethnicity, tobacco smoking, cannabis smoking, alcohol consumption, BMI, age, and sex. Estimate refers to standardized regression coefficients. Models used * to denote an interaction term. ^a^ Data were compiled from the final master file of the Québec Longitudinal Study of Child Development (1998–2015), ©Gouvernement du Québec, Institut de la statistique du Québec.

**Table 5:** Associations between adversity variables and epigenetic age acceleration, testing sex moderation in the QLSCD ^a^.

Model	Horvath	Pediatric Clock	Skin and Blood Clock	PhenoAge	GrimAge	DunedinPACE
	Estimate (*p*val)	Estimate (*p*val)	Estimate (*p*val)	Estimate (*p*val)	Estimate (*p*val)	Estimate (*p*val)
Perinatal Adversity*Sex	−0.049 (0.23)	−0.002 (0.96)	−0.021 (0.60)	0.056 (0.15)	-	0.018 (0.64)
Child and Adolescent Adversity*Sex	0.0080 (0.85)	−0.0097 (0.81)	0.0077 (0.85)	0.065 (0.11)	-	0.033 (0.40)
Perinatal Adversity*Child and Adolescent Adversity*Sex	0.05 (0.22)	−0.049 (0.24)	−0.015 (0.71)	0.051 (0.22)	-	0.073 (0.072)

Notes: All clocks, excluding DunedinPACE, were adjusted to give epigenetic age acceleration by taking the residuals of the calculated age regressed on chronological age. All age measures were additionally regressed against cell type proportions before multivariable modeling. The GrimAge Clock was also adjusted for sex before multivariable modeling, and as such was removed from sex moderation analyses. All models were controlled for self-reported race/ethnicity, tobacco smoking, cannabis smoking, alcohol consumption, BMI, and age. Estimate refers to standardized regression coefficients. In all models, sex = females. Models used * to denote an interaction term ^a^ Data were compiled from the final master file of the Québec Longitudinal Study of Child Development (1998–2015), ©Gouvernement du Québec, Institut de la statistique du Québec.

## Data Availability

Access to the data is restricted in accordance with the regulations governing data privacy in the province of Quebec (Canada), as enforced by the Institut de la statistique du Québec. As per these regulations, survey participants were assured that raw data would remain strictly confidential and would not be disclosed or shared outside a secure environment.

## Supplementary Materials

The following supporting information can be downloaded at https://www.mdpi.com/article/10.3390/biom15060887/s1: Figure S1: Cell type proportions predicted from epigenetic data in the QLSCD; Figure S2: Epigenetic age acceleration and DunedinPACE pace of aging in female and male adolescents in the QLSCD; Table S1: Summary of each covariate used in all models and their mean differences by sex in the QLSCD; Table S2: Cohen’s f^2^ for all models and measure of epigenetic age in the QLSCD; Table S3: Pearson’s correlation (r) with chronological age, mean absolute error, and maximum absolute error of the Horvath, Pediatric, Skin and Blood, PhenoAge, and GrimAge epigenetic clocks in the QLSCD.

## Abbreviations

The following abbreviations are used in this manuscript:

ELA	Early-Life Adversity
DNAm	DNA Methylation

## References

[1] McLaughlin K.A. (2016). Future Directions in Childhood Adversity and Youth Psychopathology. J. Clin. Child Adolesc. Psychol..

[2] Afifi T.O. (2020). Considerations for expanding the definition of ACEs. Adverse Childhood Experiences.

[3] Gunnar M.R. (2020). Early adversity, stress, and neurobehavioral development. Dev. Psychopathol..

[4] Boyce W.T., Levitt P., Martinez F.D., McEwen B.S., Shonkoff J.P. (2021). Genes, Environments, and Time: The Biology of Adversity and Resilience. Pediatrics.

[5] Arseneault L. (2018). Annual Research Review: The persistent and pervasive impact of being bullied in childhood and adolescence: Implications for policy and practice. J. Child Psychol. Psychiatry.

[6] Forte A., Orri M., Galera C., Pompili M., Turecki G., Boivin M., Tremblay R.E., Côté S.M. (2020). Developmental trajectories of childhood symptoms of hyperactivity/inattention and suicidal behavior during adolescence. Eur. Child Adolesc. Psychiatry.

[7] Hertzman C. (2012). Putting the concept of biological embedding in historical perspective. Proc. Natl. Acad. Sci. USA.

[8] Engert V., Efanov S.I., Dedovic K., Dagher A., Pruessner J.C. (2011). Increased cortisol awakening response and afternoon/evening cortisol output in healthy young adults with low early life parental care. Psychopharmacology.

[9] Trickett P.K., Noll J.G., Susman E.J., Shenk C.E., Putnam F.W. (2007). Attenuation of cortisol across development for victims of sexual abuse. Tissue Eng..

[10] Gunnar M.R., Vazquez D.M. (2001). Low cortisol and a flattening of expected daytime rhythm: Potential indices of risk in human development. Dev. Psychopathol..

[11] Adam E.K., Quinn M.E., Tavernier R., McQuillan M.T., Dahlke K.A., Gilbert K.E. (2017). Diurnal cortisol slopes and mental and physical health outcomes: A systematic review and meta-analysis. Psychoneuroendocrinology.

[12] Young E.S., Farrell A.K., Carlson E.A., Englund M.M., Miller G.E., Gunnar M.R., Roisman G.I., Simpson J.A. (2019). The Dual Impact of Early and Concurrent Life Stress on Adults’ Diurnal Cortisol Patterns: A Prospective Study. Psychol. Sci..

[13] Tarullo A.R., Tuladhar C.T., Kao K., Drury E.B., Meyer J. (2020). Cortisol and socioeconomic status in early childhood: A multidimensional assessment. Dev. Psychopathol..

[14] Ouellet-Morin I., Cantave C., Lupien S., Geoffroy M.C., Brendgen M., Vitaro F., Tremblay R.E., Boivin M., Côté S.M. (2021). Cumulative exposure to socioeconomic and psychosocial adversity and hair cortisol concentration: A longitudinal study from 5 months to 17 years of age. Psychoneuroendocrinology.

[15] Ioannidis K., Askelund A.D., Kievit R.A., van Harmelen A.L. (2020). The complex neurobiology of resilient functioning after childhood maltreatment. BMC Med..

[16] Weder N., Zhang H., Jensen K., Yang B.Z., Simen A., Jackowski A., Lipschitz D., Douglas-Palumberi H., Ge M., Perepletchikova F. (2014). Child Abuse, Depression, and Methylation in Genes Involved With Stress, Neural Plasticity, and Brain Circuitry. J. Am. Acad. Child Adolesc. Psychiatry.

[17] Parade S.H., Huffhines L., Daniels T.E., Stroud L.R., Nugent N.R., Tyrka A.R. (2021). A systematic review of childhood maltreatment and DNA methylation: Candidate gene and epigenome-wide approaches. Transl. Psychiatry.

[18] Provençal N., Binder E.B. (2015). The effects of early life stress on the epigenome: From the womb to adulthood and even before. Exp. Neurol..

[19] Wiechmann T., Röh S., Sauer S., Czamara D., Arloth J., Ködel M., Beintner M., Knop L., Menke A., Binder E.B. (2019). Identification of dynamic glucocorticoid-induced methylation changes at the FKBP5 locus. Clin. Epigenet..

[20] McEwen B.S. (2017). Allostasis and the Epigenetics of Brain and Body Health Over the Life Course. JAMA Psychiatry.

[21] Martin D.I.K., Cropley J.E., Suter C.M. (2011). Epigenetics in disease: Leader or follower?. Epigenetics.

[22] Oh D.L., Jerman P., Silvério Marques S., Koita K., Purewal Boparai S.K., Burke Harris N., Bucci M. (2018). Systematic review of pediatric health outcomes associated with childhood adversity. BMC Pediatr..

[23] Suarez A., Lahti J., Lahti-Pulkkinen M., Girchenko P., Czamara D., Arloth J., Malmberg A.L., Hämäläinen E., Kajantie E., Laivuori H. (2020). A polyepigenetic glucocorticoid exposure score at birth and childhood mental and behavioral disorders. Neurobiol. Stress.

[24] Bick J., Naumova O., Hunter S., Barbot B., Lee M., Luthar S.S., Raefski A., Grigorenko E.L. (2012). Childhood adversity and DNA methylation of genes involved in the hypothalamus–pituitary–adrenal axis and immune system: Whole-genome and candidate-gene associations. Dev. Psychopathol..

[25] Horvath S. (2013). DNA methylation age of human tissues and cell types. Genome Biol..

[26] Horvath S., Oshima J., Martin G.M., Lu A.T., Quach A., Cohen H., Felton S., Matsuyama M., Lowe D., Kabacik S. (2018). Epigenetic clock for skin and blood cells applied to Hutchinson Gilford Progeria Syndrome and ex vivo studies. Aging.

[27] Jain P., Binder A.M., Chen B., Parada H., Gallo L.C., Alcaraz J., Horvath S., Bhatti P., Whitsel E.A., Jordahl K. (2022). Analysis of Epigenetic Age Acceleration and Healthy Longevity Among Older US Women. JAMA Netw. Open.

[28] Johnstone S.E., Gladyshev V.N., Aryee M.J., Bernstein B.E. (2022). Epigenetic clocks, aging, and cancer. Science.

[29] Hannum G., Guinney J., Zhao L., Zhang L., Hughes G., Sadda S., Klotzle B., Bibikova M., Fan J.B., Gao Y. (2013). Genome-wide Methylation Profiles Reveal Quantitative Views of Human Aging Rates. Mol. Cell.

[30] McEwen L.M., O’Donnell K.J., McGill M.G., Edgar R.D., Jones M.J., MacIsaac J.L., Lin D.T.S., Ramadori K., Morin A., Gladish N. (2020). The PedBE clock accurately estimates DNA methylation age in pediatric buccal cells. Proc. Natl. Acad. Sci. USA.

[31] Levine M.E., Lu A.T., Quach A., Chen B.H., Assimes T.L., Bandinelli S., Hou L., Baccarelli A.A., Stewart J.D., Li Y. (2018). An epigenetic biomarker of aging for lifespan and healthspan. Aging.

[32] Lu A.T., Quach A., Wilson J.G., Reiner A.P., Aviv A., Raj K., Hou L., Baccarelli A.A., Li Y., Stewart J.D. (2019). DNA methylation GrimAge strongly predicts lifespan and healthspan. Aging.

[33] Krieger N., Chen J.T., Testa C., Diez Roux A., Tilling K., Watkins S., Simpkin A.J., Suderman M., Davey Smith G., De Vivo I. (2023). Use of Correct and Incorrect Methods of Accounting for Age in Studies of Epigenetic Accelerated Aging: Implications and Recommendations for Best Practices. Am. J. Epidemiol..

[34] Belsky D.W., Caspi A., Arseneault L., Baccarelli A., Corcoran D.L., Gao X., Hannon E., Harrington H.L., Rasmussen L.J., Houts R. (2020). Quantification of the pace of biological aging in humans through a blood test, the DunedinPoAm DNA methylation algorithm. Elife.

[35] Belsky D.W., Caspi A., Corcoran D.L., Sugden K., Poulton R., Arseneault L., Baccarelli A., Chamarti K., Gao X., Hannon E. (2022). DunedinPACE, a DNA methylation biomarker of the pace of aging. eLife.

[36] Wolf E.J., Logue M.W., Morrison F.G., Wilcox E.S., Stone A., Schichman S.A., McGlinchey R.E., Milberg W.P., Miller M.W. (2019). Posttraumatic psychopathology and the pace of the epigenetic clock: A longitudinal investigation. Psychol. Med..

[37] Mehta D., Bruenig D., Pierce J., Sathyanarayanan A., Stringfellow R., Miller O., Mullens A.B., Shakespeare-Finch J. (2022). Recalibrating the epigenetic clock after exposure to trauma: The role of risk and protective psychosocial factors. J. Psychiatr. Res..

[38] Han L.K.M., Aghajani M., Clark S.L., Chan R.F., Hattab M.W., Shabalin A.A., Zhao M., Kumar G., Xie L.Y., Jansen R. (2018). Epigenetic Aging in Major Depressive Disorder. Am. J. Psychiatry.

[39] Cerveira de Baumont A., Hoffmann M.S., Bortoluzzi A., Fries G.R., Lavandoski P., Grun L.K., Guimarães L.S., Guma F.T., Salum G.A., Barbé-Tuana F.M. (2021). Telomere length and epigenetic age acceleration in adolescents with anxiety disorders. Sci. Rep..

[40] Jovanovic T., Vance L.A., Cross D., Knight A.K., Kilaru V., Michopoulos V., Klengel T., Smith A.K. (2017). Exposure to Violence Accelerates Epigenetic Aging in Children. Sci. Rep..

[41] Lawn R.B., Anderson E.L., Suderman M., Simpkin A.J., Gaunt T.R., Teschendorff A.E., Widschwendter M., Hardy R., Kuh D., Relton C.L. (2018). Psychosocial adversity and socioeconomic position during childhood and epigenetic age: Analysis of two prospective cohort studies. Hum. Mol. Genet..

[42] Austin M.K., Chen E., Ross K.M., McEwen L.M., Maclsaac J.L., Kobor M.S., Miller G.E. (2018). Early-life socioeconomic disadvantage, not current, predicts accelerated epigenetic aging of monocytes. Psychoneuroendocrinology.

[43] Yusupov N., Dieckmann L., Erhart M., Sauer S., Rex-Haffner M., Kopf-Beck J., Brückl T.M., Czamara D., Binder E.B. (2023). Transdiagnostic evaluation of epigenetic age acceleration and burden of psychiatric disorders. Neuropsychopharmacology.

[44] Hamlat E.J., Prather A.A., Horvath S., Belsky J., Epel E.S. (2021). Early life adversity, pubertal timing, and epigenetic age acceleration in adulthood. Dev. Psychobiol..

[45] Simons R.L., Lei M.K., Klopach E., Berg M., Zhang Y., Beach S.S.R. (2021). (Re)Setting Epigenetic Clocks: An Important Avenue Whereby Social Conditions Become Biologically Embedded across the Life Course. J. Health Soc. Behav..

[46] Raffington L., Belsky D.W., Kothari M., Malanchini M., Tucker-Drob E.M., Harden K.P. (2021). Socioeconomic Disadvantage and the Pace of Biological Aging in Children. Pediatrics.

[47] Sumner J.A., Colich N.L., Uddin M., Armstrong D., McLaughlin K.A. (2019). Early Experiences of Threat, but Not Deprivation, Are Associated With Accelerated Biological Aging in Children and Adolescents. Biol. Psychiatry.

[48] Lupien S.J., McEwen B.S., Gunnar M.R., Heim C. (2009). Effects of stress throughout the lifespan on the brain, behaviour and cognition. Nat. Rev. Neurosci..

[49] Marini S., Davis K.A., Soare T.W., Zhu Y., Suderman M.J., Simpkin A.J., Smith A.D., Wolf E.J., Relton C.L., Dunn E.C. (2020). Adversity exposure during sensitive periods predicts accelerated epigenetic aging in children. Psychoneuroendocrinology.

[50] Holbrook B.D. (2016). The effects of nicotine on human fetal development. Birth Defects Res. C Embryo Today.

[51] Martins J., Czamara D., Sauer S., Rex-Haffner M., Dittrich K., Dörr P., de Punder K., Overfeld J., Knop A., Dammering F. (2021). Childhood adversity correlates with stable changes in DNA methylation trajectories in children and converges with epigenetic signatures of prenatal stress. Neurobiol. Stress.

[52] Post R.M. (2016). Epigenetic basis of sensitization to stress, affective episodes, and stimulants: Implications for illness progression and prevention. Bipolar. Disord..

[53] Bunea I.M., Szentágotai-Tătar A., Miu A.C. (2017). Early-life adversity and cortisol response to social stress: A meta-analysis. Transl. Psychiatry.

[54] Young E.S., Doom J.R., Farrell A.K., Carlson E.A., Englund M.M., Miller G.E., Gunnar M.R., Roisman G.I., Simpson J.A. (2021). Life stress and cortisol reactivity: An exploratory analysis of the effects of stress exposure across life on HPA-axis functioning. Dev. Psychopathol..

[55] McCrory C., Fiorito G., McLoughlin S., Polidoro S., Cheallaigh C.N., Bourke N., Karisola P., Alenius H., Vineis P., Layte R. (2019). Epigenetic Clocks and Allostatic Load Reveal Potential Sex-Specific Drivers of Biological Aging. J. Gerontol. Ser. A.

[56] Suarez A., Lahti J., Czamara D., Lahti-Pulkkinen M., Knight A.K., Girchenko P., Hämäläinen E., Kajantie E., Lipsanen J., Laivuori H. (2018). The Epigenetic Clock at Birth: Associations With Maternal Antenatal Depression and Child Psychiatric Problems. J. Am. Acad. Child Adolesc. Psychiatry.

[57] Engelbrecht H.R., Merrill S.M., Gladish N., MacIsaac J.L., Lin D.T.S., Ecker S., Chrysohoou C.A., Pes G.M., Kobor M.S., Rehkopf D.H. (2022). Sex differences in epigenetic age in Mediterranean high longevity regions. Front. Aging.

[58] Orri M., Boivin M., Chen C., Ahun M.N., Geoffroy M.C., Ouellet-Morin I., Tremblay R.E., Côté S.M. (2021). Cohort Profile: Quebec Longitudinal Study of Child Development (QLSCD). Soc. Psychiatry Psychiatr. Epidemiol..

[59] Silveira P.P., Pokhvisneva I., Parent C., Cai S., Rema A.S.S., Broekman B.F.P., Rifkin-Graboi A., Pluess M., O’Donnell K.J., Meaney M.J. (2017). Cumulative prenatal exposure to adversity reveals associations with a broad range of neurodevelopmental outcomes that are moderated by a novel, biologically informed polygenetic score based on the serotonin transporter solute carrier family C6, member 4. Dev. Psychopathol..

[60] Hughes M.M., Black R.E., Katz J. (2017). 2500-g Low Birth Weight Cutoff: History and Implications for Future Research and Policy. Matern. Child Health J..

[61] Radloff L.S. (1977). The CES-D Scale. Appl. Psychol. Meas..

[62] Ladd G.W., Kochenderfer-Ladd B. (2002). Identifying victims of peer aggression from early to middle childhood: Analysis of cross-informant data for concordance, estimation of relational adjustment, prevalence of victimization, and characteristics of identified victims. Psychol. Assess.

[63] Aryee M.J., Jaffe A.E., Corrada-Bravo H., Ladd-Acosta C., Feinberg A.P., Hansen K.D., Irizarry R.A. (2014). Minfi: A flexible and comprehensive Bioconductor package for the analysis of Infinium DNA methylation microarrays. Bioinformatics.

[64] Chen Y., Lemire M., Choufani S., Butcher D.T., Grafodatskaya D., Zanke B.W., Gallinger S., Hudson T.J., Weksberg R. (2013). Discovery of cross-reactive probes and polymorphic CpGs in the Illumina Infinium HumanMethylation450 microarray. Epigenetics.

[65] Pidsley R., Zotenko E., Peters T.J., Lawrence M.G., Risbridger G.P., Molloy P., Van Djik S., Muhlhausler B., Stirzaker C., Clark S.J. (2016). Critical evaluation of the Illumina MethylationEPIC BeadChip microarray for whole-genome DNA methylation profiling. Genome Biol..

[66] Zheng S.C., Breeze C.E., Beck S., Teschendorff A.E. (2018). Identification of differentially methylated cell types in epigenome-wide association studies. Nat. Methods.

[67] Lang M., Binder M., Richter J., Schratz P., Pfisterer F., Coors S., Au Q., Casalicchio G., Kotthoff L., Bischl B. (2019). mlr3: A modern object-oriented machine learning framework in R. J. Open Source Softw..

[68] McCrory C., Fiorito G., O’Halloran A.M., Polidoro S., Vineis P., Kenny R.A. (2022). Early life adversity and age acceleration at mid-life and older ages indexed using the next-generation GrimAge and Pace of Aging epigenetic clocks. Psychoneuroendocrinology.

[69] Schrempft S., Stringhini S. (2023). Socioeconomic inequalities in the Pace of Aging. Aging.

[70] Perret L.C., Geoffroy M.C., Barr E., Parnet F., Provencal N., Boivin M., O’Donnell K.J., Suderman M., Power C., Turecki G. (2023). Associations between epigenetic aging and childhood peer victimization, depression, and suicidal ideation in adolescence and adulthood: A study of two population-based samples. Front. Cell Dev. Biol..

[71] Colich N.L., Rosen M.L., Williams E.S., McLaughlin K.A. (2020). Biological aging in childhood and adolescence following experiences of threat and deprivation: A systematic review and meta-analysis. Psychol. Bull..

[72] Copeland W.E., Shanahan L., McGinnis E.W., Aberg K.A., van den Oord E.J.C.G. (2022). Early adversities accelerate epigenetic aging into adulthood: A 10-year, within-subject analysis. J. Child Psychol. Psychiatry.

[73] Yaroslavsky I., Bush A.H., France C.M. (2022). Emotion regulation deficits mediate childhood sexual abuse effects on stress sensitization and depression outcomes. Dev. Psychopathol..

[74] Luo J., Willroth E.C. (2025). Values and stress: Examining the relations between values and general and domain-specific stress in two longitudinal studies. J. Pers. Soc. Psychol..

[75] Stroud C.B., Harkness K.L., Hayden E.P. (2020). The Stress Sensitization Model. The Oxford Handbook of Stress and Mental Health.

[76] McCarthy M.M., Nugent B.M. (2015). At the frontier of epigenetics of brain sex differences. Front. Behav. Neurosci..

[77] Kundakovic M., Lim S., Gudsnuk K., Champagne F.A. (2013). Sex-specific and strain-dependent effects of early life adversity on behavioral and epigenetic outcomes. Front. Psychiatry.

[78] Aylwin C.F., Toro C.A., Shirtcliff E., Lomniczi A. (2019). Emerging Genetic and Epigenetic Mechanisms Underlying Pubertal Maturation in Adolescence. J. Res. Adolesc..

[79] Almstrup K., Lindhardt Johansen M., Busch A.S., Hagen C.P., Nielsen J.E., Petersen J.H., Juul A. (2016). Pubertal development in healthy children is mirrored by DNA methylation patterns in peripheral blood. Sci. Rep..

