# Supplementary material for: Early-Life Adversity and Epigenetic Aging: Findings from a 17-Year Longitudinal Study
Source: Biomolecules. 2025 Jun 18;15(6):887. doi: 10.3390/biom15060887 (PMC12191424; doi:10.3390/biom15060887)
Supplement: Supplementary file 1 [file biomolecules-15-00887-s001.zip › biomolecules-3682349-supplementary.pdf]

Supplemental Table 1: Summary of each covariate used in all models and their mean differences by sex in the QLSCD[a].

	Total (N=696)	Female (N=385)	Male (N = 311)	Mean Differences by Sex
	N (%)	N (%) or Mean (SD)	N (%) or Mean (SD)	*Chi-Square Test* (P-value)
Smoking	121 (17.4%)	66 (17.1%)	55 (17.7%)	0.033 (0.86)
Cannabis Consumption	126 (16.7%)	59 (15.3%)	67 (21.5%)	5.86 (0.054)
Alcohol Consumption	168 (24.1%)	87 (22.6%)	81 (26.0%)	1.27 (0.26)
White	647 (93.0%)	356 (92.5%)	291 (93.6%)	0.17 (0.68)
	Mean (SD)			*T* (P-value)
BMI	21.17 (4.01)	21.34 (4.00)	20.96 (4.01)	-0.58 (0.56)
Age	17.22 (0.27)	17.21 (0.28)	17.24 (0.27)	0.71 (0.48)

Notes: There were no mean differences by sex in the covariate measures.
[a]Data were compiled from the final master file of the Québec Longitudinal Study of Child Development (1998–2015), ©Gouvernement du Québec, Institut de la statistique du Québec.

Supplemental Table 2: Cohen's f^2 for all models and measures of epigenetic age in the QLSCD[a].

Model	Horvath	Pediatric Clock	Skin and Blood Clock	PhenoAge	GrimAge	DunedinPACE
	Cohen's f^2	Cohen's f^2	Cohen's f^2	Cohen's f^2	Cohen's f^2	Cohen's f^2
**Perinatal Adversity**	0.0074	0.026	0.019	0.057	0.013	0.098
**Child and Adolescent Adversity**	0.0086	0.029	0.018	0.057	0.014	0.096
**Perinatal Adversity + Child and Adolescent Adversity**	0.0087	0.029	0.019	0.057	0.014	0.10
**Perinatal Adversity*Child and Adolescent Adversity**	0.0096	0.029	0.019	0.061	0.012	0.10
**Perinatal Adversity*Sex**	0.0099	0.025	0.019	0.061	-	0.099
**Child and Adolescent Adversity*Sex**	0.0087	0.029	0.018	0.062	-	0.097
**Perinatal Adversity*Child and Adolescent Adversity*Sex**	0.016	0.031	0.020	0.069	-	0.11

Notes: All clocks, excluding DunedinPACE, were adjusted to give epigenetic age acceleration by taking the residuals of the calculated age regressed on chronological age. GrimAge was also controlled for sex. All models were controlled for all covariates: self-reported race/ethnicity, cigarette smoking, cannabis smoking, alcohol consumption, BMI, and age. In all models, sex = females.
[a]Data were compiled from the final master file of the Québec Longitudinal Study of Child Development (1998–2015), ©Gouvernement du Québec, Institut de la statistique du Québec.

Supplemental Table 3: Pearson's correlation (r) with chronological age, mean absolute error, and maximum absolute error of the Horvath, Pediatric, Skin and Blood, PhenoAge, and GrimAge epigenetic clocks in the QLSCD[a].

	R	Mean absolute error (years)	Maximum absolute error (years)
Horvath	0.016	3.46	9.62
Pediatric	0.033	4.66	10.26
Skin and Blood	**0.093**	5.27	8.74
PhenoAge	0.029	4.20	23.05
GrimAge	**0.077**	10.11	21.41

Notes: Bold values are significant at $p<0.05$. All measures of epigenetic age are the original values calculated by clocks prior to calculating epigenetic age acceleration. Variation in chronological age was limited in our cohort (mean = 17.24; SD = 0.43).

[a]Data were compiled from the final master file of the Québec Longitudinal Study of Child Development (1998–2015), ©Gouvernement du Québec, Institut de la statistique du Québec.

Supplemental Figure 1: Cell type proportions predicted from epigenetic data in the QLSCD[a].

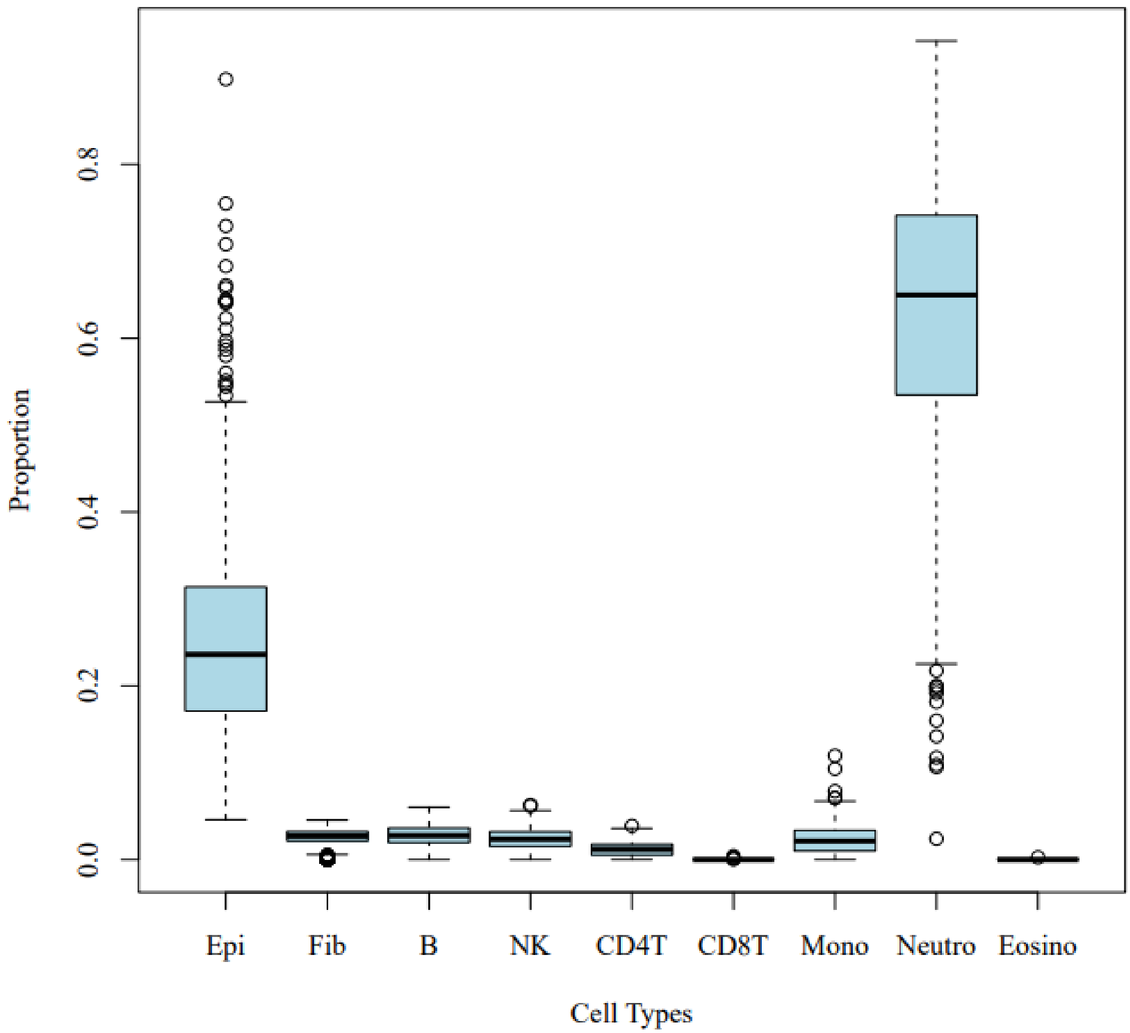

Notes: The cell types included are epithelial cells (Epi), fibroblasts (Fib), B Cells (B), natural killer cells (NK), CD4T cells (CD4T), CD8T cells (CD8T), monocytes (Mono), neutrophils (Neutro), and eosinophils (Eosino).

[a]Data were compiled from the final master file of the Québec Longitudinal Study of Child Development (1998–2015), ©Gouvernement du Québec, Institut de la statistique du Québec.

Supplemental Figure 2: Epigenetic age acceleration and DunedinPACE pace of aging in female and male adolescents in the QLSCD[a].

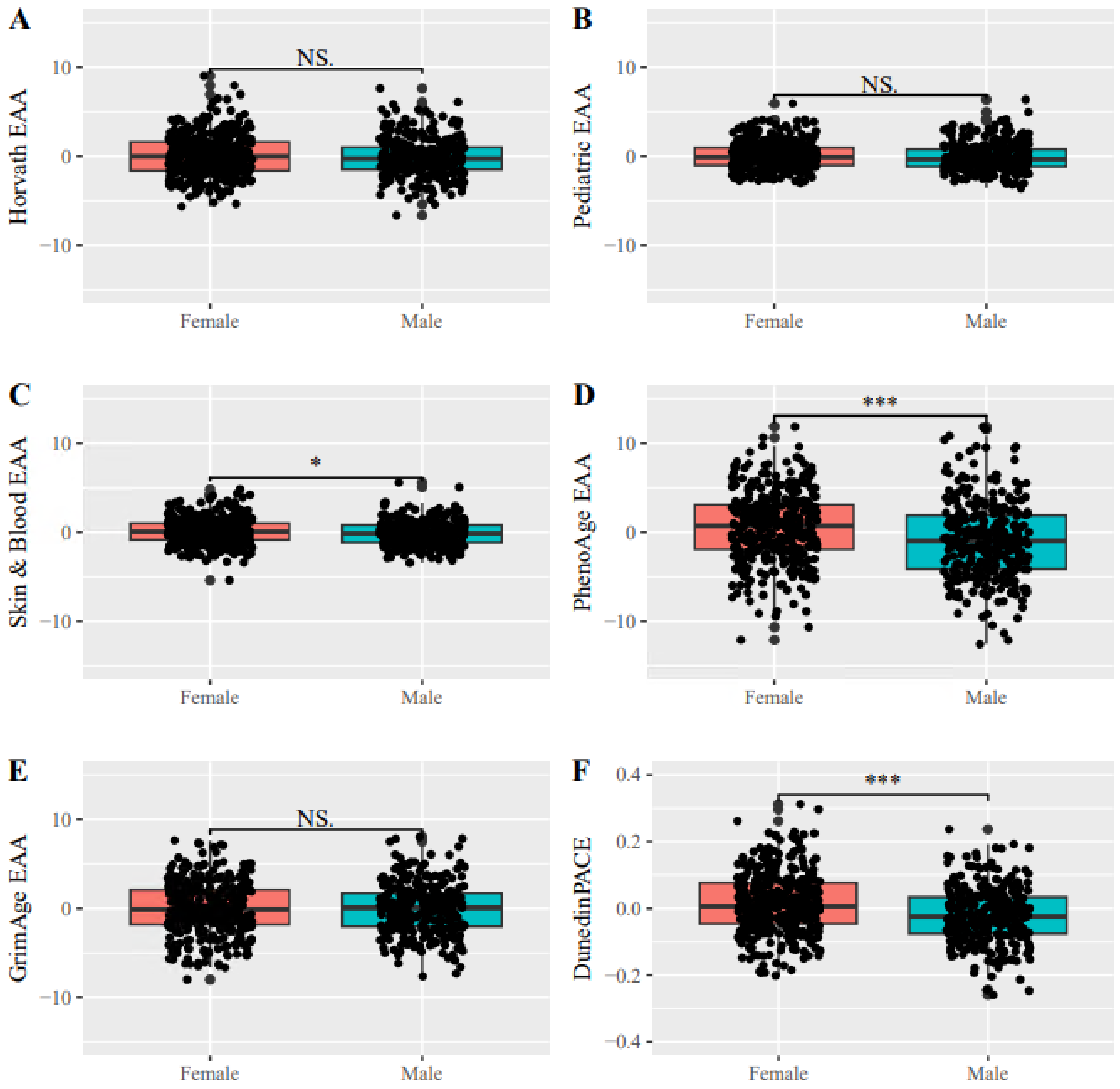

Notes: All clocks (A-E) display epigenetic age acceleration: (A) Horvath Clock, (B) Pediatric Clock, (C) Skin and Blood Clock, (D) PhenoAge Clock, (E) GrimAge Clock, and (F) DunedinPACE pace of aging.
[a]Data were compiled from the final master file of the Québec Longitudinal Study of Child Development (1998–2015), ©Gouvernement du Québec, Institut de la statistique du Québec.
